# Effect of the Combination of Superabsorbent Polymers for Autogenous Shrinkage Control with Steel Fibers of High-Performance Concrete under Uniaxial Tension Using DIC

**DOI:** 10.3390/ma13204638

**Published:** 2020-10-17

**Authors:** Karyne Ferreira dos Santos, António Carlos Bettencourt Simões Ribeiro, Eugênia Fonseca da Silva, Manuel Alejandro Rojas Manzano, Leila Aparecida de Castro Motta, Romildo Dias Toledo Filho

**Affiliations:** 1 Postgraduate Program in Structural Engineering and Construction–PECC, Department of Civil and Environmental Engineering, SG-12 Building, University of Brasilia–UnB, 70910-900 Brasília-DF, Brazil; eugenia@unb.br; 2 Materials Department, Nacional Laboratory for Civil Engineering–LNEC, Av. do Brasil 101, 1700-075 Lisbon, Portugal; bribeiro@lnec.pt; 3 Department of Civil and Industrial Engineering, Faculty of Civil Engineering and Science, Pontifical Xavierian University of Cali, Calle 18 N° 118-250, Edificio Central, 2do. Piso, Cali 760031, Colombia; alejandro.rojas@javerianacali.edu.co; 4 Faculty of Civil Engineering–FECIV, Federal University of Uberlândia–UFU, Av. João Naves de Ávila, 2121, Santa Mônica Campus 1Y, 38400-902 Uberlândia-MG, Brazil; lacastro@ufu.br; 5 The Alberto Luiz Coimbra Institute for Graduate Studies and Research in Engineering–COPPE, Department of Civil Engineering, University of Rio de Janeiro–UFRJ, Av. Horácio Macedo 2030, Ilha do Fundão, 21941-972 Rio de Janeiro, RJ, Brazil; toledo@coc.ufrj.br

**Keywords:** superabsorbent polymer, steel fibers, high-performance fiber reinforced concrete, digital image correlation, autogenous shrinkage, tensile behavior

## Abstract

This paper presents a study of the effect of a superabsorbent polymer (SAP) for autogenous shrinkage control on the uniaxial tensile behavior of steel fiber reinforced concrete (SFRC). The use of fibers and SAP potentially increases the durability of the concrete, preventing cracking by autogenous shrinkage and enhancing post-cracking behavior. Furthermore, SAP can provide further hydration for self-healing purposes and improve the ductility of the SFRC. In order to evaluate the effect of the addition of SAP in SFRC, dog-bone SFRC specimens with different dosages of superabsorbent polymers were cast and tested under uniaxial tension. The digital image correlation (DIC) technique was used to understand the effect of SAP on the steel fibers’ crack-bridging mechanisms. Surface strains and crack openings were inferred using the DIC technique. The effect of SAP and fibers on fresh and hardened concrete was individually investigated by flow tests and compressive strength tests. Autogenous shrinkage was measured in plain concrete to investigate the minimum SAP content required to mitigate autogenous shrinkage of 0.3%. The use of 0.3% SAP was also sufficient to reach multiple cracking behavior. This content of SAP completely suppressed the autogenous shrinkage with minimal side effects on compressive strength. An analytical formulation for the tensile behavior of SFRC was developed using the variable engagement model, presenting a mean correlation of R^2^ of 0.97 with the experimental results.

## 1. Introduction

The present investigation focused on the tensile behavior of steel fiber reinforced concrete (SFRC) with superabsorbent polymer (SAP). In general, SAP is used to control autogenous shrinkage without significantly affecting strength and workability [1,2,3,4,5,6,7,8,9]. Fibers are used in concrete for cracking control [10,11,12,13,14,15].

The synergic effect of SAP and fibers has been mainly studied with synthetic fibers [16,17,18,19,20,21,22]. The studies of concrete with synthetic fibers and SAP shows that the presence of the two constituents modifies the tensile behavior of the composite, and improvements on the durability of the material can be achieved [23,24,25]. The improvements are due to the reduction in the size of the crack by increasing the number of cracks. This multi-cracking behavior is obtained not only from the presence of fibers, but also by the multi-flaws provided by SAP [16].

Studies of concrete with fibers and SAP are scarce. The study presented by [26] showed promising results on the use of SAP to densify the interfacial transition zone and reduce micro-cracks around the fibers. Wang et al. [27] conducted a splitting tensile test study with SAP and steel fiber for cellular concrete applications, although the concrete was not high strength. None of these studies were performed with concrete direct tension.

The present document intends to contribute to the understanding of the overall behavior of the composite subjected to direct tension by analyzing the crack formation and crack pattern with the digital image analysis technique, which allows for simultaneously observing the behavior of a set of fibers and flaws produced by the SAP addition, and the interaction between them. This type of behavior cannot be observed when testing only one fiber in tension [26] nor with the splitting test [27].

The investigation also deals with other specific subjects: (1) Investigate the maximum SAP content to be incorporated in high strength concrete (HSC) and steel fiber reinforced concrete (SFRC) regarding the loss of compressive strength and workability; (2) Investigate a minimum SAP content that controls the autogenous shrinkage of HSC; (3) Characterize the tensile properties of SFRC with different SAP contents and complement the regular analysis with the crack pattern with full-field strain measurement using DIC; and (4) Develop an analytical model for predicting the tension behavior of HSC with varying SAP dosage.

## 2. Materials and Methods

### 2.1. Materials

Portland cement of high initial strength conforming to [28] Type CPV-ARI (CIPLAN, Brasília, Brazil) was used for all mixes in this study. A silica fume, of the non-densified type, meeting the requirements of the standard [29] was supplied by the national company Silmix (Breu Branco, Pará, Brazilcountry). The physical and chemical properties of the cement and silica fume are shown in Table 1. Locally available sand of the Corúmba River, with the maximum size of 4.75 mm and gradation conforming to [30] standard usable zones, was used. The sand fineness module was 2.73, and the specific mass was 2.65 kg/dm³. A water reducer of high-efficiency, superplasticizer ADVA CAST 129 from Grace Company (Sorocaba, São Paulo, Brazil), based on polycarboxylates, was used to maintain the fluidity of the mortar within a fixed range for all mixes.

#### 2.1.1. Steel Fibers

**Figure 1:**
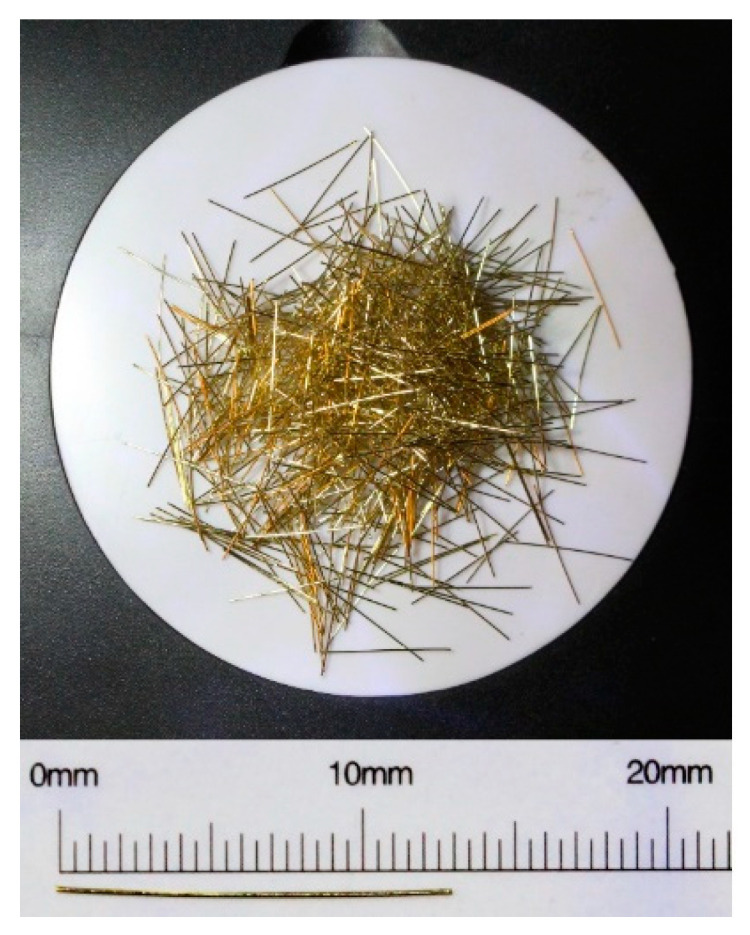
Dramix Ol 13/.16 steel fiber from Bekaert Company.

Steel fibers of DRAMIX OL 13/.16 mm from BEKAERT (Zwevegem, Belgium), illustrated in Figure 1, with the content of 1.28% in volume, were used. These were made of smooth steel of 13 mm in length, 0.16 mm in nominal diameter, aspect ratio of 81.25, Young’s modulus of 200 GPa, and tensile strength of 2750 MPa.

#### 2.1.2. Superabsorbent Polymers (SAP)

**Figure 2:**
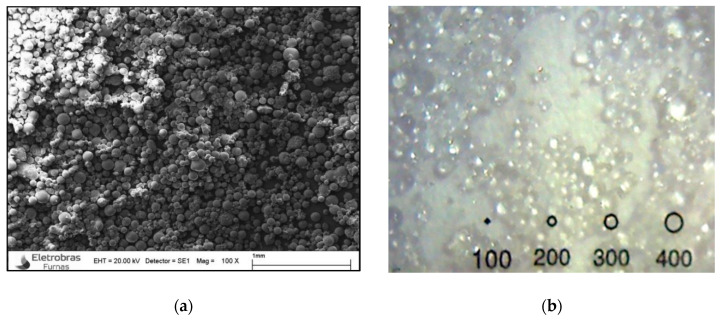
Superabsorbent polymer: (**a**) dry; (**b**) swollen. The spheres have a diameter in micrometers.

The superabsorbent polymer used was an acrylic acid/acrylamide (Technical University of Denmark, Lyngby, Denmark) with covalent cross-links produced by the reverse suspension polymerization technique, with a mean particle size (D_50_) of 66.3 µm. It has an absorption in the cementitious medium of 18 g of water for 1 g of SAP, and the density of 1.456 g/cm³. The particle size curve of the SAP characterized by the portion of particles with diameters smaller than 27.3 µm is 10% (D_10_), and the portion of particles with diameters below 101.7 µm is 90%. (D_90_). It was developed for particular use in a high alkaline environment such as the cement suspension. It was supplied by Prof. Ole Mejlhede Jensen and developed at the Technical University of Denmark (DTU). In Figure 2, the polymer is presented in the dry and swollen state by using the scanning electron microscope and digital microscope H1000X / S1000X, respectively.

The water absorption capacity of SAP was measured by the slump flow consistency method and by means of graduated cylinders proposed by [31]. Absorption of SAP was tested for deionized water and cement filtrate since the absorption of the mixing water by the SAP takes place with the addition of water to the remaining components of the concrete mix, in a way that the activation of SAP takes place in a highly active electrochemical environment. The absorption capacity in deionized water was 96 g of water per 1 g of SAP.

The smaller absorption of cement filtrate, when compared to absorption of water, caused by calcium and magnesium ions present in the cementitious fluid (increase the cross-linking in SAP) [32,33], is an advantage for self-healing purposes. This effect leads to a smaller void creation in the hardened mortar and, after crack formation, when the SAP gets into contact with clean water, it will have swollen larger and temporarily seal the crack. The saturated environment can then provoke self-healing of the crack by hydration of the anhydrous cement under certain conditions and quantities of SAP.

#### 2.1.3. Mix Proportions

In order to investigate the combined effect of the SAP and steel fibers, eight concrete mixtures were prepared according to Table 2. These included three reference compositions, two without SAP or fibers (REF-035 and REF-040), and another with fibers and without SAP (REF-035F). The remaining five mixtures included SAP or SAP and fibers.

All mixtures presented low w/c ratio and high strength. The total water–cement (w/c) ratio was set at 0.35 or water–binder of 0.32. A reference mixture with w/c of 0.4 was also prepared for comparison with the mixture where additional water was placed due to SAP. In the mixtures without fibers, the content of the superplasticizer was adjusted in order to reach the required workability.

The mortar compositions all had the same proportions of binder and aggregate and the content of SAP varied from 0 to 0.6%. In the mixtures with fibers, the amount of superplasticizer was fixed at 2% and the fibers content was set constant at 1.28% in volume.

The mortars were prepared in a mechanical mixer (Solotest São Paulo, Brazil) under the following steps. The first step was dry mixing the cement, the SAP, and the sand for 5 min at a slow speed. The second step was the water and superplasticizer addition for 2 min, and then mixing at a high speed for 5 min. Then, the mixer was stopped, the edges of the bowl were scraped, and followed by mixing for another 3 min at high speed. In the correspondent mixtures, the fibers were then added constantly in motion for 5 min at slow speed, preventing the ball bearing effect, then the edges were scraped again for 3 min. Finally, the mortar was mixed for 5 min at high speed to ensure a uniform consistency. For proper compaction, the mixes were cast into molds placed on a vibrating table. The specimens were moved to a room with 100% relative humidity and 22 ± 1 °C. They were demolded after 24 h and continued in this room until the testing date.

### 2.2. Experimental Program

#### 2.2.1. Measurement of Flowability and Compressive Strength

Slump flow tests were carried out right after mixing, following the procedure [34]. The fresh mortar was poured into a Hagerman cone (larger base diameter of 100 mm, height of 60 mm, and a smaller base of 70 mm) into two layers. Each layer was tamped 10 times evenly distributed, and then the cone was gently removed, and the spread was measured. The largest diameter was measured along with the diameter in the perpendicular direction.

Compressive tests were carried out according to the standard [35]. Cylinder specimens (Ø 50 mm × L 100 mm) were used, and an average of three tested samples was reported. The base of the specimens was ground to ensure a smooth and plane surface. The test adopted a Microcomputer-controlled electromechanical universal test systems (MTS), and the loading rate was 0.5 mm/min.

#### 2.2.2. Autogenous Shrinkage

**Figure 3:**
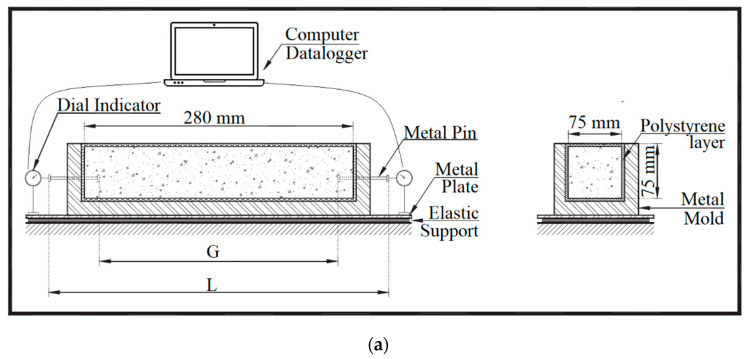

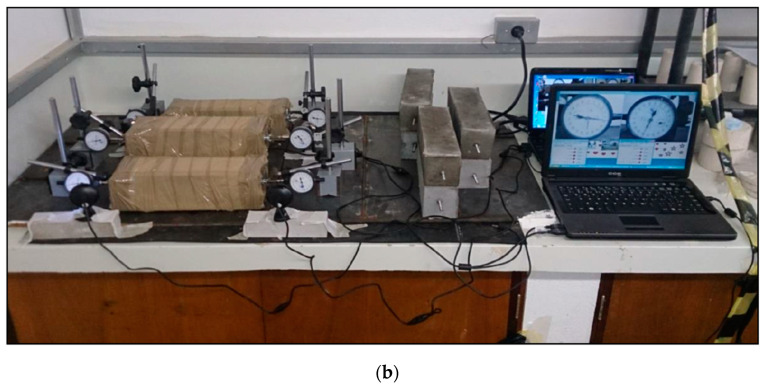
Autogenous shrinkage test: (**a**) test setup scheme and (**b**) on going test.

The autogenous shrinkage was assessed according to the methodology developed by [36], which is a modified method of [37] using the [38] apparatus, shown in Figure 3. The test consists of the measurement of the deformation of mortars, cast in a prismatic mold with nominal dimensions of 7.5 × 7.5 × 28.5 cm. Each mold was previously prepared, with polystyrene layers inside to decrease friction, allowing the concrete to move freely. Additionally, two threaded metal pins were placed in the extremities of the mold aligned with the specimen’s longitudinal axis. Strain gauges are couples in these pins and connected to a computer to collect and store the data. The distance between these pins is called G, and the measurement of this distance determines the shrinkage.

After casting, to prevent the loss of water to evaporation, the mold with the mortar was wrapped with multiple layers of polystyrene and adhesive tape. The weight of the mold with mortar was measured, and they were stored in a chamber with 50 ± 2% of humidity and 21 ± 2 °C for 28 days, where the straingauges continuously recorded the G distance.

#### 2.2.3. Uni-Axial Testing Equipment with Digital Image Correlation (DIC) Arrangement

**Figure 4:**
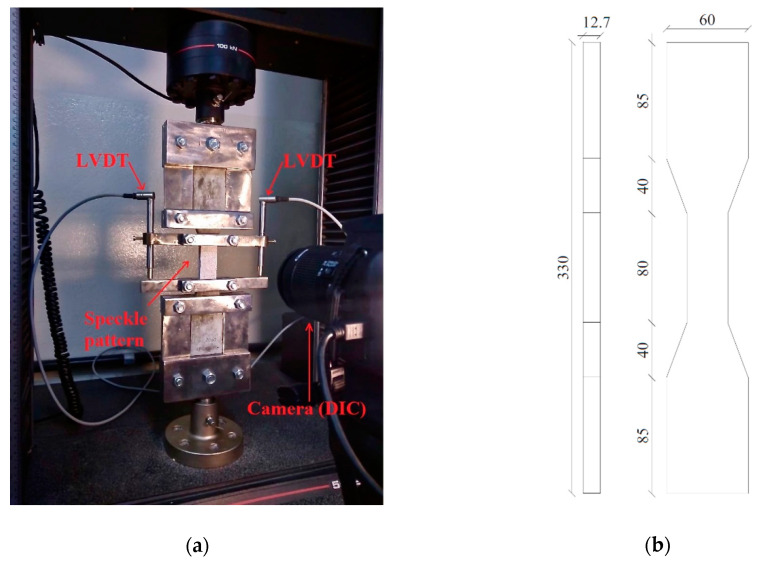
The direct uniaxial tensile test: (**a**) test setup with DIC and (**b**) specimen dimension (mm).

In order to verify the strain-hardening behavior of all HPC mixtures at 28 days, a series of direct tensile tests were performed following [39]. An INSTRON (Instron, São José dos Pinhais, Paraná, Brazil) electronic universal testing machine with displacement control and load capacity of 100 kN was used at a constant speed of 0.3 mm/min. The loading force was measured on a computerized data recording system as for the strain was measured by two linear variable displacement transducers (LVDT, HBM, São Paulo, Brazil), placed on both sides of the specimen. Additionally, the strain was also measured by digital image correlation (DIC, Correlated Solutions, Irmo, United States of America). The tensile setup and geometry of the specimen are shown in Figure 4.

The DIC was used to analyze the crack pattern and the continuous deformation of the specimen concerning the applied load to assist the investigation of the strain hardening behavior with the addition of the SAP and steel fibers. The DIC setup included a digital camera, sufficient light in the specimen, and the sample preparation. The sample preparation consisted of painting the specimen with white paint and aleatory and heterogeneously painting dark dots to form distinct patterns that can be recognized by the image correlation program. For the program to work well, these dots, the camera configuration, and position were adjusted for each dot to have four to six pixels in the picture. Before the test began, a calibration image was taken for each test to convert the pixel scale to a millimeter scale. The camera, testing machine, and LVDT were all started simultaneously so that the data could be correlated later on.

Image processing software VIC-2D was used in this study to correlate different images and the corresponding deformations. The software relates the deformed images by dividing the area of interest (AOI) into many small regions, called subsets, where each subset is unique and identified by the program via the dots pattern. The program detects the change in the first image subset, set as a reference, with the images taken during the test and calculates the distance, which is used to calculate the full-field displacement and strains by interactive techniques [40].

Due to the brittle behavior, it is not possible to obtain the strain–stress curve of plain concrete specimens with the direct tension test with displacement control. In this case, the splitting tensile strength test was carried out based on [41]. This method was used mainly to evaluate the effect of SAP incorporation on tensile strength. Three cylindrical samples (Ø 100m × 200 mm) were used to measure the splitting tensile strength of concrete on the seventh and 28th day. The machine for testing the splitting tensile strength was a MTS microcomputer-controlled electromechanical universal test systems, with the loading speed of 0.2 MPa/s.

## 3. Results and Discussions

### 3.1. Influence on Mortar Flow

The flow values of each mix and the corresponding superplasticizer content are presented in Table 2. The results of mixture REF-35 and SAP-02, without fibers, showed that to keep the same flow (183 mm) in both mixtures, 0.2% of SAP required an increase of 12% in the superplasticizer dosage. Mixture SAP-0.3, with the same superplasticizer dosage but another 0.1% of SAP, led to a reduction in the flow (180 mm). This effect on flow is expected, as the increase of SAP content also increased the particle concentration, but the effect was limited and manageable. Paiva [42] proposed that a water-reducing agent could be efficient at maintaining the flowability since SAP particles do not interfere with the plasticizer chains.

The adverse effects of steel fibers on the workability of concrete have been widely discussed by [43], and reinforced by recent publications with high strength concrete such as [44,45]. In order to have good workability after the incorporation of fibers, the superplasticizer content was increased to 2% of the cement weight, which led to a reference flow of 307 mm (REF-035F).

The effect of SAP on the flow can be more clearly seen in the last four mixtures of Table 2, since the water content and the superplasticizer dosage were kept constant. The flow progressively decreases as the addition of SAP increases. The reduction was 20.85%, 25.1%, and 32.2% for 0.2%, 0.3%, and 0.6% of SAP, respectively.

The decrease in the workability suggests that the additional water provided to fill the SAP is actually being absorbed by the SAP, and the flow decrease is due to lesser free water per unit of volume. Some extra water absorption by the SAP may also be occurring, these findings were supported by [46]. The opposite effect was reported by [47,48,49], which leads to a gap in the literature as the effect of the SAP in the workability is not entirely understood. The authors in [4,31,50] explained that the broad diversification of results would depend on the methodology used to accurately estimate the amount of water absorbed by SAP in the cementitious environment. The over or underestimated amount of additional water can affect the workability and the total w/c. Another hypothesis of the loss of workability was provided by [51], who believed that the swollen SAP particles behaved as soft aggregates and offered a restraining effect in the rheology of the mortar. Nevertheless, all the mixtures maintained good workability and no signs of segregation.

### 3.2. Compressive Strength

**Figure 5:**
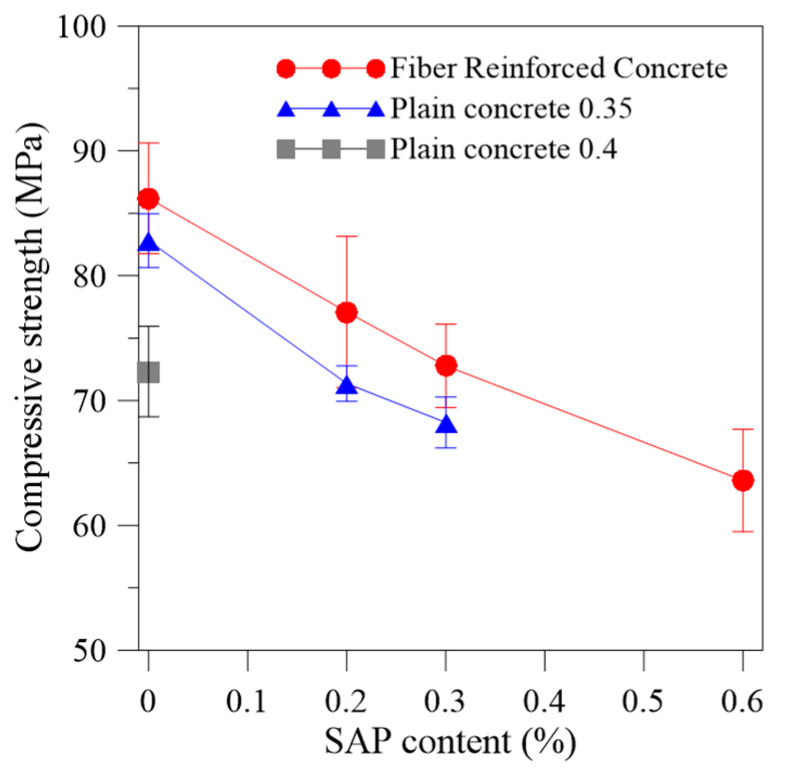
Compressive strength results at 28 days of the fiber reinforced concrete of the w/c_(basic)_ of 0.35; plain concrete without fiber reinforcement with the w/c_(basic)_ of 0.35, and the plain concrete without fiber reinforcement with the w/c_(basic)_ of 0.40.

A summary of the compressive strength results for the fiber reinforced concrete and the plain concrete at 28 days is provided in Figure 5 and Table 3. Each compressive strength result is the average of six specimens.

Increases in SAP dosage for the same w/c_(basic)_ tended to almost linearly decrease the compressive strength for both mixture series (with and without fiber reinforcement). However, comparing the compressive strength of mixtures with same w/c_(total)_, REF-040, and SAP-0.3, the values were similar. This indicates the major role of the total volume of pores on strength, regardless of the presence or absence of SAP.

Strictly speaking, in order to individually evaluate the influence of the SAP, a specific reference mixture should be manufactured containing the same total w/c ratio, but for the purposes of the present work, this information was not considered necessary.

This subject leads to a discussion presented in the literature that has yet to be enlightened. Many authors have reported the loss of compressive strength in the literature. However, as published by [50], this loss of strength could be provoked by the excess of water addition due to a misleading measure of the SAP absorption in the cementitious environment. Simple methods have been used to estimate the SAP absorption capacity and could be overestimating the water of absorption, increasing the water in the mixture, and lowering the compressive strength. However, this hypothesis was not validated by the slump results obtained in this research. If there were an excess of water in the fresh state, the slump would not decrease since it would facilitate the workability.

Another explanation and more common for the loss of strength, supported by [4,51,52,53], and others, determined that the initial swelling of SAP creates a reasonable amount of macropores due to the SAP swelling. Snoeck et al. [51] further explained that in the fresh mix, macropores spontaneously form and become occupied by swollen SAP particles. Following this, the concrete pore solution is consumed by cement hydration, which decreases the ambient moisture where the SAP is located. Afterward, SAP slowly releases the inside water, causing the SAP to shrink. After SAP voids form, they result in increases in the total porosity of the concrete system. However, some studies have reported a straight gain due to effective internal curing, where the later hydration of the cement provided by the SAP entrapped water densified the pore structure, which was not the case in this study.

The addition of steel fibers in the mixture increased the compressive strength in 4.11%, 8.03%, and 5.51% when compared with the reference without fiber reinforcement and w/c_(basic)_ of 0.35 for the 0.2%, 0.3%, and 0.6% of SAP incorporation, respectively. The 0.6% of SAP was determined to be the highest SAP incorporation for this mix design. Given that, according to [54,55], the lower limit of strength to be classified as HSC is 55 MPa, for this research, it was stipulated to reach a minimum value of 60 MPa so that the concrete can be classified as high strength.

### 3.3. Autogenous Shrinkage

The deformation measured in the test was considered as autogenous shrinkage since minimum moisture exchange occurred between the specimens and environment due to the coat of aluminum and plastic tape applied to the specimens before starting the test.

**Figure 6:**
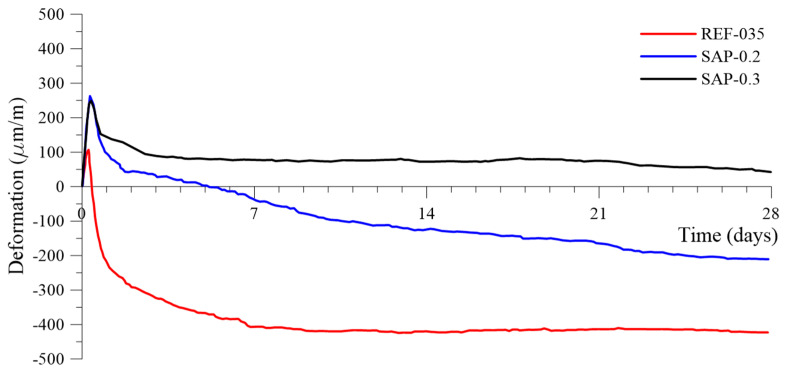
Autogenous strain (um/m) for cement mortar mixtures without fibers w/c_(total)_ of 0.35, with SAP additions of 0.2% and 0.3%, determined from time zero up to 28 days.

The scope of the research was to find the content of SAP that could mitigate or control autogenous shrinkage. Therefore, the worst-case scenario was to carry out the test without fiber reinforcement. The autogenous shrinkage for the mixtures without fiber reinforcement up to 28 days are shown in Figure 6. Each value represents an average of three specimens. As displaced in Figure 6, the reference, REF-035, presented autogenous shrinkage much higher than that of ordinary concrete, and increased significantly in the first seven days due to the absence of coarse aggregate and low water/binder ratio. REF-035 presented a maximum deformation of 424 µm/m and an initial expansion of 106 µm/m, which was overcome by autogenous shrinkage after 10 h.

The general trends of the curves in Figure 6 tended to be stable after 10 days. When studying the SAP-containing mixtures, the percentage of 0.3% completely mitigated the autogenous shrinkage and presented a maximum expansion of 248 µm/m at six hours after setting. The shrinkage did not counterbalance the expansion and, after 28 days, still presented 42 µm/m of expansion. The expansion phenomenon is not yet fully understood, but there are several attempts at explanation, for example, involving expansive pressure by forming hydration products (the high MgO content of the cement used may be a source of early expansion). This can be beneficial for some prestressed applications since the concrete compressive strength is higher than its tensile strength. The material is likely to withstand the maximum compression efforts induced by the expansion. Additionally, this expansion can be helpful and contribute to preventing cracking from drying shrinkage.

The content of 0.2% of SAP addition reduced 90% of the autogenous shrinkage compared to the reference at the age of seven days and reduced 50% at 28 days. It also presented an expansion of 262 µm/m at four hours after time 0. The authors in [1,2,56] described the water releasing mechanism of the SAP after setting of the cement-based material, which explained the reduction of autogenous shrinkage. The incorporation of SAP leads to the formation of controlled water-filled microscope inclusions, which prevent internal moisture evaporation from compensating water loss for curing, promote the hydration of unhydrated cement, and reduce the autogenous shrinkage.

The use of more than 0.3% of SAP addition is considered to ensure autogenous shrinkage control, with beneficial properties such as the expansion, which can avoid cracking and produce a more durable concrete, as seen in [57].

### 3.4. Tensile Properties

For the unreinforced specimens, the splitting tensile test was performed, and the tensile strength with the ratio of tensile to compressive strength for all mixtures is presented in Table 3. The specimens failed as expected, releasing almost all the energy soon after the peak load. The average tensile strength of the specimens without fiber reinforcement was 5.11 ± 0.2 MPa.

**Figure 7:**
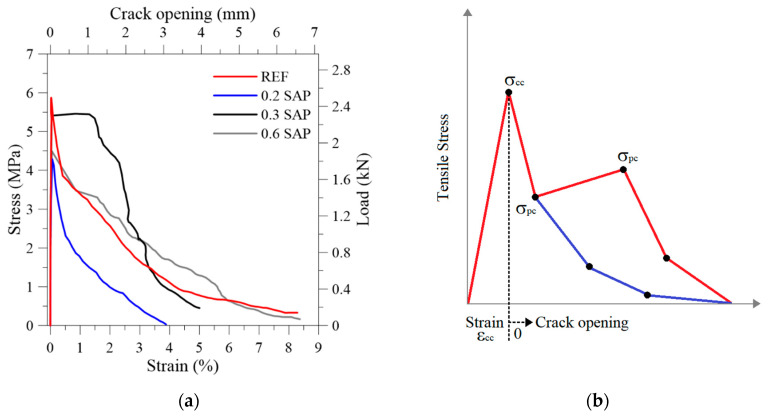
Stress x strain curve of: (**a**) SFRC with varying SAP content; (**b**) typical curves of strain softening behavior.

Typical stress-strain/load-displacement curves of the developed SFRC with SAP particles at 28 days are presented in Figure 7a. A diagrammatic sketch of the strain-softening behavior presented by [10] is shown in Figure 7b, who classified the composites based on their tensile response. The parameters regarding Naaman (2006), chosen to characterize the tensile behavior and to implement the analytical model described in the next section, were: first structural cracking stress (σ_cc_) and force (F_cc_); first structural cracking strain (ε_cc_) and displacement (d_cc_); maximum post-cracking stress (σ_pc_) and force (F_pc_); crack opening (w_pc_); and tension toughness index (TTI), as presented in Table 4. Before the crack opening, the acquired displacement was calculated as a strain of the composite; after the first crack, it was evaluated as a crack opening.

**Figure 8:**
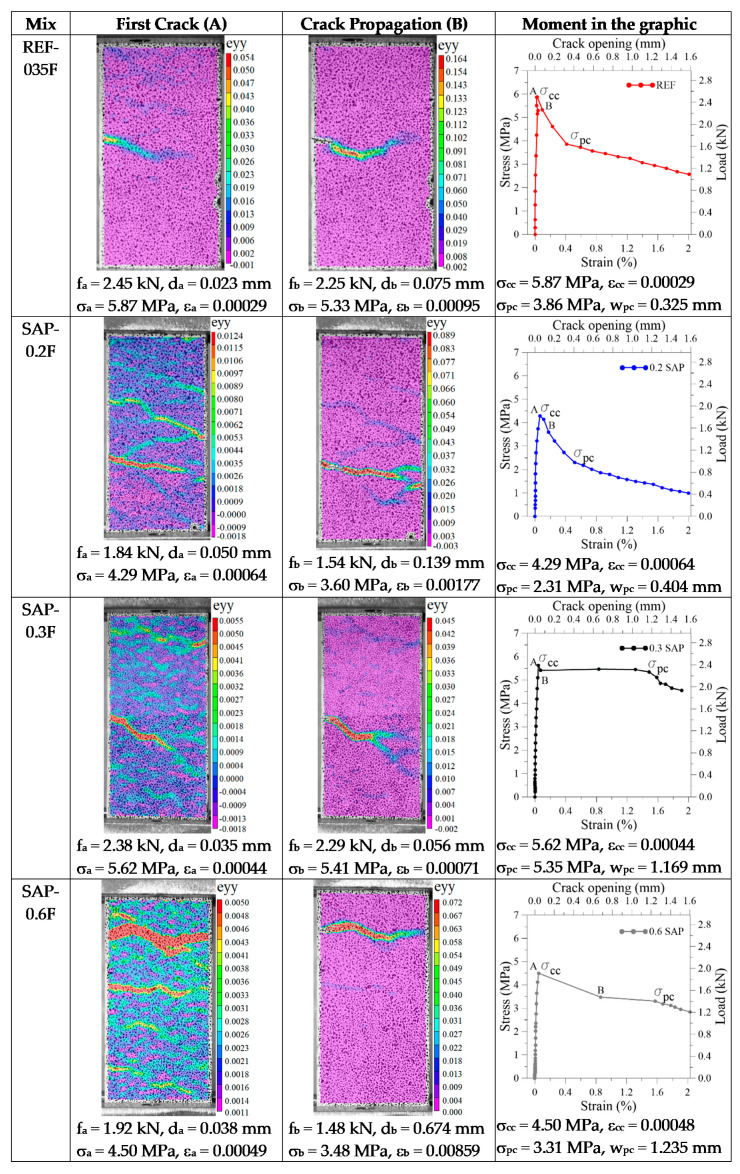
Morphology of cracking apparent on the face of a specimen of SFRC, where A is the first cracking point, and B is the crack propagation, both presented in the respective graphic for each mix.

The main aspect to be witnessed by the SAP incorporation was the increase in the ductility of the composite. This behavior could be better observed by the DIC analysis shown in Figure 8 as the crack pattern of the SFRC. According to [10], the higher the strength, the lower the strain at the peak stress. This phenomenon could be observed in this experiment. Therefore, the general trade-off that exists in most materials between strength and ductility also applies to the developed composites. The tension toughness index (TTI) is a measurement of toughness calculated by area under the stress × strain curve until the specimen ultimately failed. In general, higher tension toughness (or energy absorption) was tightly related to higher SAP content, except for the SAP-0.2F. Compared to the reference, the TTI for SFRC increased by 21% for SAP-03F, 12% for SAP-06F, and decreased 37% for SAP-02F.

From Figure 8, the images of the DIC in the first cracking point were named A. The point soon after the cracking occurrence was named B. These points were correlated between the camera and the data acquisition. It was possible to observe that at the first cracking stress (σ_cc_), point A, the composite without SAP produced one transverse localized crack and propagated later on at point B. As for the composites with SAP, they produced a different damage mode, leading to the creation of several cracks in the first peak stress (σ_cc_). Moreover, when the stress concentration opens a single crack, the cracking evolution, shown in point B, produces a different morphology with more branches, releasing more energy and in accordance with the TTI results.

The authors in [16] incorporated SAP to control and improve the performance of fiber-reinforced concrete with polyvinyl alcohol (PVA) fibers. SAP improved the ductility of the material by the insertion of a mechanical flaw. According to the micromechanical theory developed by [24,25], one of the criteria to increase the toughness and multiple cracking in the cementitious composite is when σ_cc_ ≤ σ_pc_. This criterion can be achieved by decreasing the strength of the matrix by inserting a flaw in the matrix. The matrix tensile strength equals the stress of the bond between the fiber and matrix, and the composite develops a more ductile behavior.

This behavior was intensely studied with PVA fibers. However, for steel fibers, it was observed that as soon as the specimen cracked on reaching the σ_cc_, there was a sudden drop in stress resistance, leading to extensive cracking, widening before the stresses were transferred from the matrix to the fibers (σ_pc_). Nevertheless, it was also noted from Figure 8 that the SAP incorporation enhanced the toughness of the composite as it increased the multiple-crack behavior when the difference of σ_cc_ and σ_pc_ was lower. The 0.3% SAP addition achieved the best behavior because the regain of strength after the first cracking was significant. The difference between the two stresses was 0.3 MPa. However, for the developed SFRC, the σ_pc_ was not higher than the σ_cc_, and the multiple-cracking behavior was not achieved. Still, the fibers fulfilled their purpose to increase the toughness and produce a progressive, yet gradual decrease in the load-carrying capacity.

The overall trend of the tensile strength presented a decrease as the SAP addition increased. The mixture with a content of 0.2% SAP was the exception. Despite following the general tendency to decrease the tensile strength by the insertion of SAP, it presented, in both cases of the concrete with and without fiber reinforcement, a higher reduction than the ones with more SAP insertion. This behavior was not expected and no reasonable explanation was found since, for the results of compressive strength and slump, it was inside the pattern of decrease. As for the autogenous shrinkage, it also presented results inside the expected trend.

Liu, Farzadnia and Shi [27] along with Wang et al. [26], are the few articles investigating the tensile behavior of steel fiber reinforced concrete with superabsorbent polymers. Wang et al. [27] focused on investigating different steel fibers types and contents with an established SAP content in the mixture, as opposed to our research that fixed the fiber type and content to investigate the influence of different SAP additions.

As reported by [26], the addition of SAP increased the flexural to compressive strength ratio of their ultra-high performance concrete (UHPC). For the UHPC with small size SAP, the increase in the ratio regarding the reference was 8% with 0.3% of SAP and 22% with 0.6% of SAP. Despite the difference in the tensile measurement, flexural strength is an indirect measure of the tensile strength. This phenomenon could also be observed for the concrete without fiber reinforcement (Table 3). The ratio increased by 9% for the addition of 0.2% SAP and 14% for the increase with 0.3%. The increment in the tensile to compressive strength ratio means that the SAP’s internal cure is more beneficial for the development of tensile than compressive strength. Liu, Farzadnia and Shi [26] raised the hypothesis that the addition of SAP increased the interstitial bonding strength of steel fibers. Additionally, this could be one of the critical factors in increasing the tensile to compressive results. However, for SFRC, only the 0.3% of SAP addition showed an improvement in the ratio of 16%; as for the other content, no improvement was observed.

#### Analytical Tensile Evaluation of SAP Incorporation

There are many analytical models to describe the behavior of fiber reinforced concrete, especially regarding steel fiber reinforcement, for example, [58,59,60]. It was not within the scope of this work to evaluate the best analytical model to describe the experimental obtained curves. Instead, a more contemporary and consolidated model was chosen to describe and predict the SFRC. The variable engagement model (VEM) has been extensively used to investigate fiber reinforcement, even in DIC analyses of direct tensile test such as in [61], more details of the model can be found in [62,63]. This model gives an approach for modeling strain softening behavior on uniaxial tension, where the fibers are randomly orientated in three dimensions.

**Figure 9:**
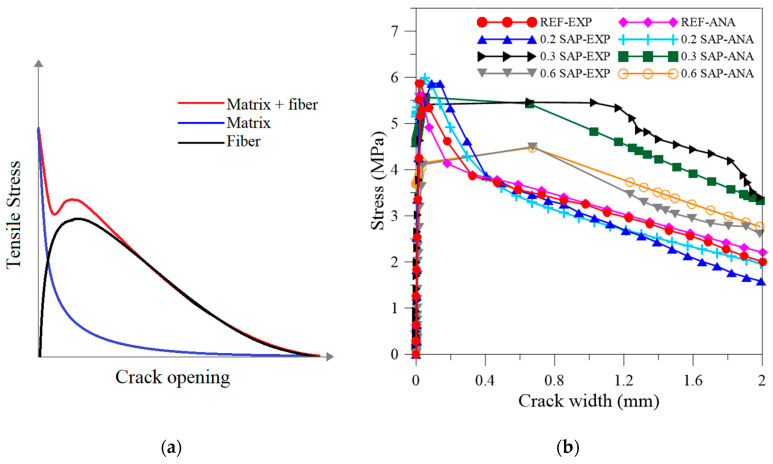
(**a**) Typical stress versus crack opening after cracking for the fiber–matrix composite. (**b**) Analytical model with experimental results for the SFRC with SAP additions.

For modeling the developed SFRC, the fibers were straight, so the mechanical anchorage was dismissed for the model. The total tensile stress developed by the model was composed by the sum of the stress of the matrix and the stress provided by the frictional bond between fiber and matrix, as exemplified in Figure 9a and Equation (1).

(1) $$f_{t}=f_{ct}+f_{st}$$

where *f_t_* is the total tension stress carried by the fiber reinforced concrete; *f_ct_* is the stress carried by the matrix; and *f_st_* is the stress carried by the fiber.

VEM considers that the embedded fiber is pulled out from the crack’s side with the shorter embedded fiber length, and ignores the axial elastic deformation of the fibers. An exponential tension softening relationship is provided as:

(2) $$f_{ct}=f_{t}^{\prime }\cdot \exp\left(-c\cdot w\right)$$

where *f’_t_* is the tensile strength of the specimens without fibers; *w* is the crack width of the specimen at a given load; and *c* is an attenuation factor for the concrete matrix undergoing tension decay after cracking. The *c* parameter was a variable to the model fitting into the experimental curves.

The stress carried by the fiber is given by:

(3) $$f_{st}=K_{f}\alpha_{f}\rho_{f}\tau_{b}$$

where *K_f_* is the global orientation factor; *α_f_* is the aspect ratio; *ρ_f_* is the volumetric ratio; and *τ_b_* is the mean shear stress between the fiber and the matrix.

The aspect ratio is given by:

(4) $$\alpha_{f}=\frac{l_{f}}{d_{f}}$$

where *l_f_* is the length of the fiber and *d_f_* the diameter, and the global orientation factor is given by:

(5) $$K_{f}=\frac{\left(\tan^{-1}\left(w/\alpha \right)\right)}{\pi }{\left(1-\frac{2w}{l_{f}}\right)}^{2}$$

where *α* is the engagement parameter, which for fiber composites with straight or end hooked steel fibers is of *α* = *d_f_*/3.5.

For the model to work without the fiber fracture, the following equation must be satisfied. For the present case, it was fulfilled, and the model without fiber fracture was chosen. Additionally, by observing the cracked section after the tensile test was performed, no fiber fracture was observed.

(6) $$l_{f}<l_{c}=\frac{d_{f}}{2}\frac{\sigma_{fu}}{\tau_{b}}$$

where *l_c_* is the critical fiber length and *σ_fu_* is the ultimate tensile strength of the fiber.

The analytical model presented using Equations (1)–(6) was plotted with the experimental results in Figure 9b, where EXP designates the experimental and ANA represents the analytical curves.

Since the composites presented the same fiber content and type, in order for the model to fit the experimental results, only three parameters could vary: the parameter c that is dependent of the type of concrete; the stress of the fiber bond; and the tensile stress of each composite at 28 days. The tensile stress was provided by the results obtained. The other parameters were initially based on the literature and then modified to adjust the model to the experimental curves better.

The fitting of these parameters revealed that the bond between the fiber and matrix was enhanced with the SAP addition, except for the 0.2% as already discussed, to eventually be an out layer. The initial value set for the bond strength was 10 MPa, tested by [64]. The interpolation to better fit the experimental curve to the model presented the following results: for the reference, the stress bond was 9 MPa, and SAP-0.2F, SAP-0.3F, and SAP-0.6F were 8, 13.5, and 11.2 MPa, respectively. This confirmed that SAP-0.3F presented a superior bond between the fiber and matrix, consequently enhancing the toughness and presenting the best behavior. Furthermore, SAP-0.6F validated that the SAP addition enhanced the bond between the matrix and steel fiber.

The parameter c can be physically interpreted as the behavior of the concrete or mortar undergoing tension decay after cracking; the typical value of concrete is 15 and 30 for mortar. Furthermore, the interpolation revealed that the SAP addition modified the matrix to be more likely to be a mortar than concrete. The REF-035 was 15 as a result of the fitting, which is the typical value of concrete. The addition of 0.2, 0.3, and 0.6 presented the values of 6, 18, and 22, respectively.

Overall, the proposed models presented a good correlation, indicated by a mean correlation R^2^ of 0.97. Future work to investigate the pull-out behavior and confirm the indicated values should be performed. Furthermore, the analytical model can describe the behavior of the SFRC with SAP and can be applied in the future analysis of elements under uniaxial tension.

## 4. Conclusions

This study established the framework to investigate the tension behavior of a durable steel fiber reinforced concrete with increasing dosage of SAP and to develop a HSC with SAP to mitigate autogenous shrinkage and develop an analytical model for predicting the tension behavior of HSC with varying SAP dosage. Based on the results presented, the following conclusions can be drawn:

- HSC can incorporate a dosage of 0.6% of SAP, keeping high strength and workability.
- Autogenous shrinkage can be adequately mitigated with the content of 0.3% of SAP. However, a content of 0.2% may give sufficient reduction to avoid cracking.
- The content of 0.3% of SAP was shown to be beneficial in different aspects. It successfully mitigated autogenous shrinkage, can be used without major influence on workability, is compatible with high compressive strength, and significantly enhanced the SFRC’s ductile performance.
- The variable engagement model used was capable of describing the behavior of the SFRC with SAP. Moreover, it can be used in future finite element applications.

## Figures and Tables

**Table 1:** Basic chemical composition of cement and silica fume.

Component	Cement (%)	Silica Fume (%)
SiO_2_	24.41	93.95
Al_2_O_3_	7.09	0.16
CaO	53.74	0.74
Fe_2_O_3_	3.02	0.27
MgO	4.36	0.86
SO_3_	3.28	-
Na_2_O	0.28	0.37
K_2_O	0.77	0.84
Density (g/cm^3^)	3.03	2.21

**Table 2:** Composition of the concrete mixtures, values in kg/m^3^.

Mixture (kg/m^3^)	Cement	Silica	Sand	SAP	Fiber	Superplasticizer	w/c_(basic)_	w/c_(total)_^1^	Flow (mm)
REF-035	675.8	67.6	1306.5	0	0	11.15	0.35	0.35	183
REF-040	654.2	65.4	1264.5	0	0	8.50	0.40	0.40	200
SAP-0.2	675.8	67.6	1306.5	1.35	0	12.50	0.35	0.38	183
SAP-0.3	675.8	67.6	1306.5	2.03	0	12.50	0.35	0.40	180
REF-035F	675.8	67.6	1306.5	0	100	13.50	0.35	0.35	307
SAP-0.2F	675.8	67.6	1306.5	1.35	100	13.50	0.35	0.38	243
SAP-0.3F	675.8	67.6	1306.5	2.03	100	13.50	0.35	0.40	230
SAP-0.6F	675.8	67.6	1306.5	4.06	100	13.50	0.35	0.46	208

^1^ Including water absorbed by SAP.

**Table 3:** Mechanical properties of the high strength concrete and steel fiber reinforced concrete.

Mixture	Mean Compressive Strength (MPa)	Strength Reduction Considering the w/c_(basic)_ (%)	Mean Tensile Stress (MPa–First Cracking	Ratio of Tensile to Compressive Strength
REF-035	82.80 ± 2.1	-	5.39	0.065
REF-040	72.32 ± 3.6	-	4.92	0.068
SAP-0.2	71.36 ± 1.4	13.82	5.04	0.071
SAP-0.3	68.24 ± 2.0	17.58	5.07	0.074
REF-035F	86.20 ± 4.4	-	6.01 ± 0.45	0.070
SAP-0.2F	77.09 ± 4.8	10.57	4.59 ± 0.25	0.060
SAP-0.3F	72.78 ± 4.8	16.47	5.92 ± 0.04	0.081
SAP-0.6F	63.60 ± 4.1	26.22	3.23 ± 0.23	0.051

**Table 4:** Tensile properties of SFRC specimens at 28 days.

Mixture	F_cc_ (kN)	F_pc_ (kN)	σ_cc_ (MPa)	σ_pc_ (MPa)	d_cc_ (mm)	ε_cc_ (%)	w_pc_ (mm)	TTI (MPa)
REF-035F	2.45	1.63	5.87	3.86	0.023	0.029	0.325	0.1336
SAP-0.2F	1.85	0.99	4.29	2.31	0.050	0.064	0.404	0.0491
SAP-0.3F	2.38	2.26	5.62	5.35	0.035	0.044	1.169	0.1623
SAP-0.6F	1.92	1.41	4.50	3.31	0.038	0.048	1.235	0.1491
